# TRIM33 promotes glycolysis through regulating P53 K48-linked ubiquitination to promote esophageal squamous cell carcinoma growth

**DOI:** 10.1038/s41419-024-07137-z

**Published:** 2024-10-10

**Authors:** Tian Xia, Lian Meng, Guixuan Xu, Hao Sun, Hao Chen

**Affiliations:** 1 College of Basic Medical Sciences, Chongqing Medical University, Chongqing, 400016 China; 2 Department of Pathology and Key Laboratory for Xinjiang Endemic and Ethnic Diseases, The First Affiliated Hospital, Shihezi University School of Medicine, Shihezi, 832002 China; 3 Department of Pathology, Sichuan Provincial People’s Hospital, School of Medicine, University of Electronic Science and Technology of China, Chengdu, 610072 China; 4 Department of Critical Care Medicine, Huazhong University of Science and Technology Union Shenzhen Hospital (Nanshan Hospital), Shenzhen, 518052 China

**Keywords:** Tumour biomarkers, Tumour-suppressor proteins

## Abstract

Esophageal squamous cell carcinoma (ESCC) is a common fatal malignant tumor of the digestive tract; however, its pathogenic mechanism is unknown and lacks specific molecular diagnosis and treatment. Therefore, it is particularly important to identify new tumor biomarkers to enhance the early diagnosis and molecular-targeted therapy of ESCC. Here, we found that the E3 ubiquitin ligase Tripartitemotif-containing33 (TRIM33) is highly expressed in ESCC tissues and cell lines, and is associated with adverse clinical outcomes. We determined that TRIM33 drives aerobic glycolysis to promote tumor growth in vivo and in vitro. In terms of mechanism, TRIM33 binds to p53 to inhibit its stability and promote the expression of downstream glycolysis target genes GLUT1, HK2, PKM2, and LDHA. In addition, TRIM33 promotes the polyubiquitination of P53 K48-linked and proteasome degradation. Further studies have shown that the K351 site of P53 is the key site mediating the ubiquitination of P53 K48-linked to promote aerobic glycolysis in ESCC and tumor cell growth. Our results reveal that the TRIM33-P53 signal axis regulates glycolysis during ESCC and may provide a new perspective for the diagnosis and treatment of ESCC.

## Introduction

Esophageal squamous cell carcinoma (ESCC) is a common malignant tumor of the digestive tract, ranking seventh among all malignant tumors and sixth in terms of overall mortality [1]. Although progress has been made in diagnosis and treatment, the overall five-year survival rate of patients with esophageal squamous cell carcinoma worldwide remains less than 30% [2, 3]. Therefore, there is an urgent need for new tumor biomarkers to enhance the early diagnosis and molecular-targeted therapy of ESCC and improve the survival rate of patients with ESCC.

Metabolic reprogramming affects cancer occurrence and development. Even when there is a large amount of oxygen in cancer cells, the reprogramming of glucose metabolism mainly involves glycolysis. Aerobic glycolysis (Warburg effect) has been shown to be the main driver of cancer [4]. Activation of the Warburg effect in cancer cells has been recognized as a marker of cancer, and its metabolites are necessary for cancer cell proliferation and tumor progression. Therefore, characterizing the cooperative mechanisms of glycolysis and cell proliferation could improve our understanding of human cancer development. Some literatures have shown that TRIM23, a member of the E3 ubiquitin ligase TRIM family, acts as an oncogene in lung adenocarcinoma (LUAD) and regulates glucose metabolism through the TRIM23/NF- κ B/GLUT1/3 axis to promote DDP drug resistance [5]. TRIM46 activates AKT/HK2 signal transduction by modifying PHLPP2 ubiquitin to promote glycolysis and chemotherapy resistance in cancer cells [6]. Therefore, we were interested in the role of TRIM family members in metabolic remodeling and its potential mechanism.

TRIM33, a member of the E3 ubiquitin ligase family, is involved in a wide range of biological processes, including DNA repair, cell differentiation, inflammation, and cancer [7]. Cancer studies have shown that TRIM33 acts as an oncogene in the development of several tumors. In B cell lymphoblastic leukemia, pancreatic cancer, cervical cancer, and prostate cancer [8–11], TRIM33 can promote tumor growth and prevent tumor cell apoptosis. In addition to its tumor-promoting function, the expression of TRIM33 was downregulated in non-small cell lung cancer, breast cancer, glioma, and renal clear cell carcinoma [12–15]. Although abnormal expression of TRIM33 has been shown to play an important role in regulating biological processes and tumorigenesis, the role and mechanism of TRIM33 in the progression of ESCC, its function in regulating the complex metabolic reprogramming of ESCC, and the molecular mechanism of TRIM33 as an E3 ubiquitin ligase are still unclear.

In this study, we confirmed the clinical correlation between TRIM33 and ESCC, and clarified the high expression of TRIM33 in ESCC. In vivo and in vitro experiments confirmed that TRIM33 promotes the occurrence and development of ESCC. Mechanistically, TRIM33 interacts with P53 to promote aerobic glycolysis and esophageal cancer by inducing P53 K48-linked proteasome-dependent ubiquitin to inhibit P53. In general, these results reveal the important role of TRIM33-mediated post-translational modifications in aerobic glycolysis and tumorigenesis and provide a theoretical basis for the molecular therapy of ESCC.

## Results

### TRIM33 is highly expressed in ESCC tissues and cell lines, and is associated with adverse clinical outcomes

**Fig. 1:**
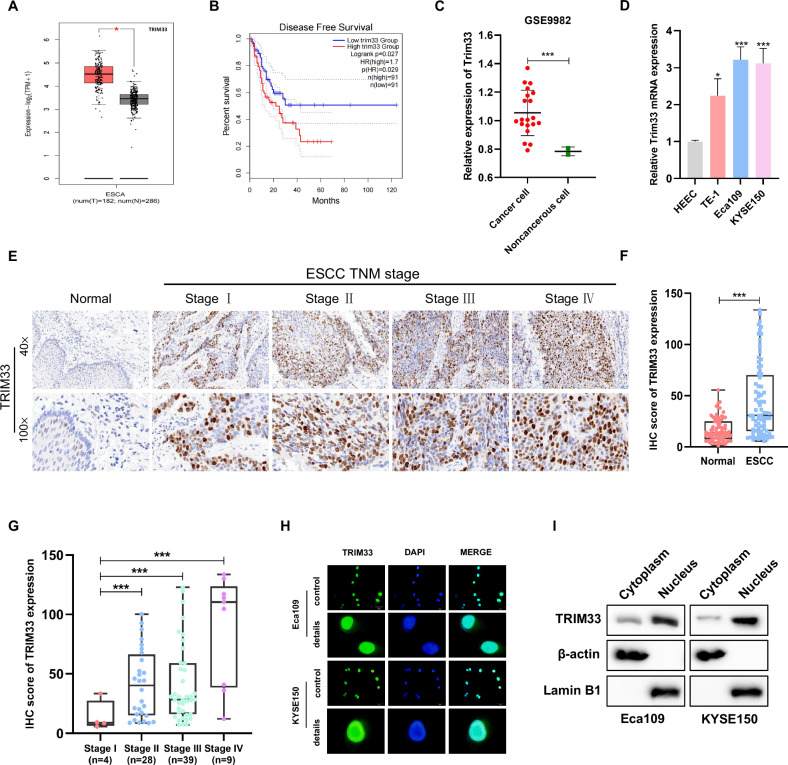
Upregulation of TRIM33 in ESCC. **A** TRIM33 expression in normal tissues and ESCA tissues from TCGA databases. **B** Analysis of relapse-free survival of patients in TCGA dataset. **C** GEO data (GSE9982) analysis of TRIM33 mRNA expression in normal esophageal cell lines (*n* = 2) and esophageal cancer cell lines (*n* = 20). **D** TRIM33 mRNA expression in human normal esophageal cells and ESCC cells. **E** IHC staining of TRIM33 expression in human ESCC (clinical stages I–IV) and normal esophageal tissues. **F** The quantitative analysis of TRIM33 expression in ESCC and normal esophageal tissues. **G** The quantitative analysis of TRIM33 expression in human ESCC (clinical stages I–IV). **H** Immunofluorescence staining revealed that the METTL5 protein was mainly localized in nucleoli. Scale bars, 50 μm. **I** Western blot for TRIM33 expression in cytoplasmic and nuclear fractions of ESCC cells, The nuclear internal reference Lamin B and the cytoplasmic internal reference β-actin were used to detect the separation efficiency.

To explore and verify the expression of TRIM family members in esophageal cancer, we first analyzed the expression of TRIM in normal esophageal tissues and esophageal cancer tissues in The Cancer Atlas (TCGA) database and found that 11 TRIM family members were highly expressed in esophageal cancer compared with normal tissues. Next, we studied whether the expression levels of these 11 genes were related to disease-free survival (DFS) in patients with esophageal cancer. Our analysis of esophageal cancer data from TCGA through GEPIA (http://gepia.cancer-pku) showed that only a high level of TRIM33 was closely related to poor DFS (*P* = 0.027), while the other ten TRIM family members showed no significant correlation (Figs. 1A, B and S1). The GSE9982 dataset from the public Gene Expression Omnibus (GEO) database showed that the expression of TRIM33 in the esophageal cancer cell line was higher than that in normal cells (Fig. 1C). Consistent with the published data, quantitative real-time polymerase chain reaction (qRT-PCR) analysis showed that the level of TRIM33 mRNA in ESCC cell lines (TE-1, Eca109, and KYSE150) was higher than that in normal esophageal epithelial HEEC cells (Fig. 1D). In addition, we analyzed the expression of TRIM33 in 80 patients with ESCC and 79 normal esophageal tissues by immunohistochemical staining with TRIM33 antibody. The quantitative results showed that the expression of TRIM33 in ESCC was higher than that in normal esophageal tissues, and the staining site was mainly in the nucleus, which may be related to the tumor grade (Stage IV > Stage III ≈ Stage II > Stage I) (Fig. 1E–G). The immunofluorescence and nuclear-cytoplasmic separation results showed that TRIM33 was mainly located in the nucleus, with low expression in the cytoplasm (Fig. 1H, I). Overall, these results indicated that TRIM33 is highly expressed in ESCC and may be a valuable clinical biomarker in patients with ESCC.

### TRIM33 plays the role of an oncogene in ESCC

**Fig. 2:**
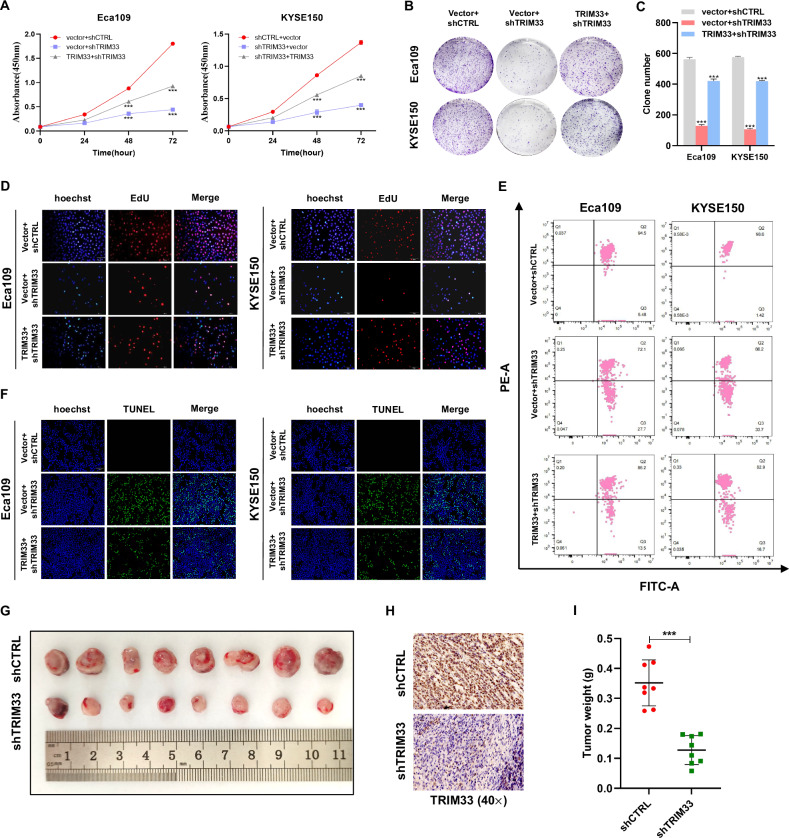
TRIM33 is required for cancer cell growth and apoptosis resistance in ESCC. **A**–**D** Knockdown of TRIM33 by shRNA suppresses the proliferation of ESCC cells, but the restoration of TRIM33 expression partially rescued the negative effect of shTRIM33 on cell proliferation as revealed by the CCK-8 assay (**A**), colony formation assay (**B**, **C**), and EdU assay. Scale bars, 50 μm (**D**). **E** The effect of TRIM33 expression on the membrane potential of Eca109 and KYSE150 cells was analyzed by JC-1 assay. **F** Overexpression of TRIM33 partially decreased the number of TUNEL-positive cells induced by the TRIM33 knockdown. Scale bars, 100 μm. **G** Xenograft model in nude mice; representative images of tumors from all mice in each group. **H** Representative images of IHC staining of TRIM33 in Mice tumor tissues. **I** Mice tumor weights.

To study the potential function of TRIM33 in the development of ESCC, we silenced the expression of endogenous TRIM33 in Eca109 and KYSE150 cells with lentiviral delivery of shRNAs and used a recombinant plasmid containing the pEnCMV-TRIM33-Myc gene to establish Eca109, KYSE150, and TE-1 cells overexpressing TRIM33. The expression level of TRIM33 was detected by western blotting and qRT-PCR (Fig. S2A, B). The results of the CCK-8 cell viability, colony formation, and EdU proliferation assays showed that the downregulation of TRIM33 significantly inhibited the growth and colony formation of ESCC cells. Rescue assays were performed on this basis. The results showed that the overexpression of TRIM33 partially saved the negative effect of shTRIM33 on cell proliferation (Fig. 2A–D). We then evaluated the effect of TRIM33 on cell death. The results of membrane permeability JC-1 staining showed that, compared to the control group, shTRIM33 decreased the mitochondrial membrane potential, indicating that shTRIM33 promoted depolarization of the mitochondrial membrane and provided a basis for the early apoptosis of ESCC cells, which could be reversed by the overexpression of TRIM33 (Fig. 2E). TUNEL assays showed that shTRIM33 treatment increased the number of TUNEL-positive apoptotic cells, whereas TRIM33 overexpression partially reduced the number of TUNEL-positive cells induced by TRIM33 knockdown (Fig. 2F). Similarly, silencing TRIM33 in TE-1 cells can inhibit cell proliferation and promote cell death (Fig. S2C–E). These data suggest that TRIM33 promotes ESCC cell growth. using stable shTRIM33-Eca109 cells, we studied the role of TRIM33 in vivo by establishing a xenotransplantation mouse model. Our data showed that deletion of TRIM33 inhibited tumor growth in vivo (Fig. 2G–I). In summary, these data support a tumor-promoting role of TRIM33 in ESCC.

### TRIM33 promotes aerobic glycolysis in ESCC

**Fig. 3:**
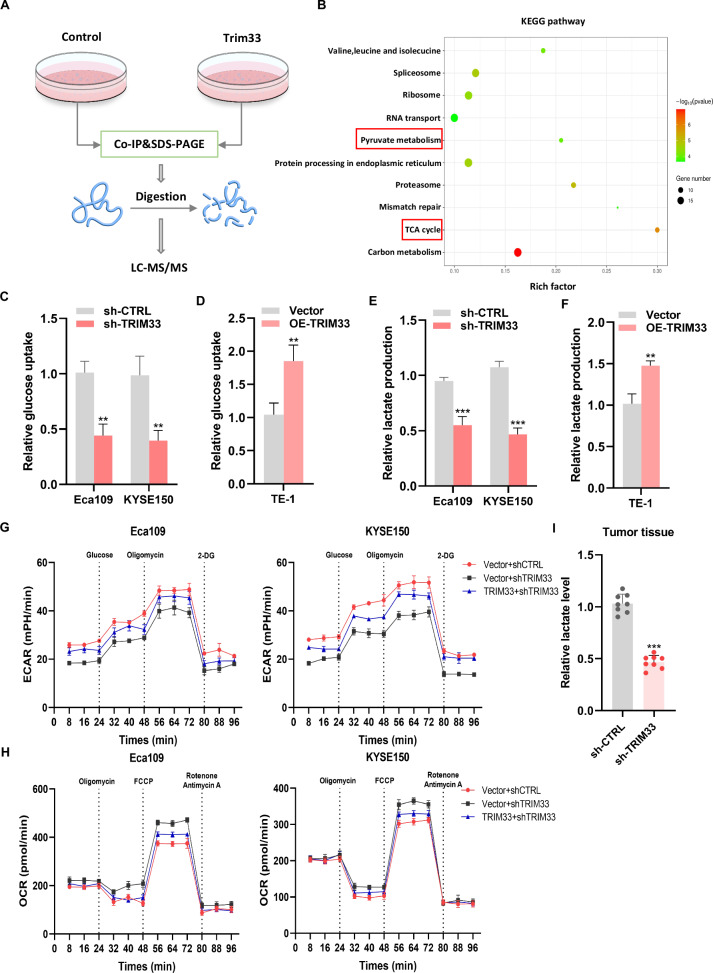
TRIM33 enhances aerobic glycolysis of ESCC cells. **A** The flow chart of Co-IP of TRIM33 and subsequent LC-MS/MS combined analysis. **B** KEGG pathway analysis of LC-MS/MS data. **C**–**F** After knocking down or overexpressing TRIM33, the glucose uptake rates (**C**, **D**) and lactic acid production (**E**, **F**) in three different cell lines were detected. **G** The influence of TRIM33 on the extracellular acidification rate (ECAR) of Eca109 and KYSE150 cells was measured by an XF extracellular flux analyzer. **H** Seahorse assays to monitor cellular oxygen consumption rate (OCR) in ESCC cells. **I** The changes in lactic acid content in tumor tissues of nude mice after knocking down TRIM33 were detected.

To further explore the mechanism by which TRIM33 participates in ESCC progression, we combined Co-IP and LC-MS/MS to identify potential proteins interacting with TRIM33 (Fig. 3A) and performed bioinformatic analysis of these proteins. KEGG enrichment analysis showed that the potential target protein of TRIM33 affected a variety of signal pathways related to tumor metabolism, such as pyruvate production and TCA cycle related to glucose metabolism (Fig. 2B and S3A). GO enrichment analysis revealed various biosynthetic processes (Fig. S3B). Therefore, we suspected that TRIM33 promotes ESCC growth by affecting glycolysis. We measured glucose uptake and lactic acid production after silencing TRIM33 to systematically study whether TRIM33 activation promotes aerobic glycolysis (the Warburg effect) in ESCC. This glycolytic phenotype is inhibited by glucose absorption and reduced lactic acid production. As expected, TRIM33 silencing significantly reduced glucose uptake and lactic acid production, whereas TRIM33 overexpression had the opposite effect (Fig. 3C–F). We subsequently performed Seahorse assays to monitor the intact cellular extracellular acidification rate (ECAR) and oxygen consumption rate (OCR) in living cells, and silencing TRIM33 significantly impaired ECAR in ESCC cells, however, OCR levels significantly increased, which could be reversed by restoring TRIM33 expression (Fig. 3G, H). This indicates that ESCC cells undergo a metabolic shift from oxidative phosphorylation to glycolysis, which is consistent with the Warburg effect. Lactic acid detection in mouse tumor tissues showed that the expression level of lactic acid in the shTRIM33 group was lower than that in the control group (Fig. 3I). In summary, these results suggested that TRIM33 promotes ESCC growth by promoting aerobic glycolysis.

### TRIM33 promotes aerobic glycolysis of ESCC by inhibiting P53

**Fig. 4:**
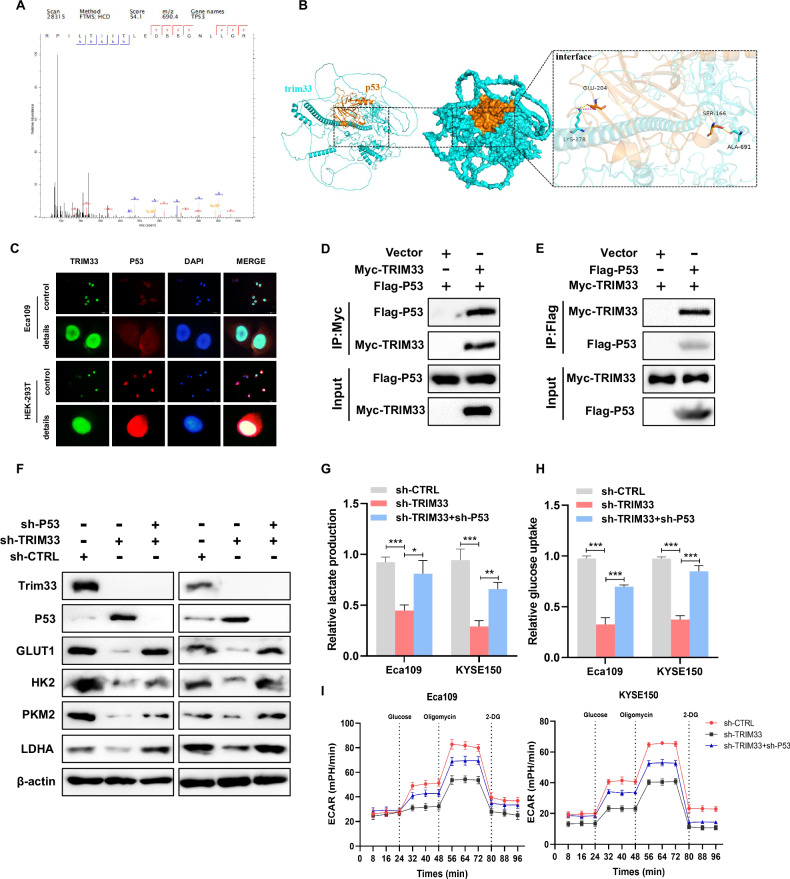
TRIM33-P53 axis plays an important role in glucose metabolism. **A** Peptide fragment mass spectrum of P53 protein. **B** Protein docking map for predicting the interaction between TRIM33 and P53. **C** Fluorescence microscope analysis showed that TRIM33 and P53 were co-located in ESCC cells. Scale, 50 μm. **D**, **E** Co-IP analysis in HEK293 cells confirmed the interaction between TRIM33 and P53. **F** Protein expression level of glycolysis-related genes in ESCC cells with different treatments. **G**–**I** Glucose uptake (**G**), lactic acid production (**H**), and ECAR (**I**) were measured in ESCC cells after different treatments.

To further clarify the potential molecular mechanism by which TRIM33 promotes aerobic glycolysis in ESCC, based on the results of IP and LC-MS analyses, we identified P53 (Fig. 4A), a tumor suppressor molecule. In most cases, P53 can inhibit glycolysis through multiple steps. Protein docking analysis showed that TRIM33 and P53 share a structural motif for direct binding. The predicted results from Piper revealed that TRIM33 and P53 may form a protein–protein interaction interface (Fig. 4B). To confirm the specific interaction between TRIM33 and P53, immunofluorescence detection showed that TRIM33 and P53 were mainly colocalized in the nucleus (Fig. 4C). In addition, we analyzed the expression of TRIM33 and P53 in 80 patients with ESCC by immunohistochemical staining and performed correlation analysis. The results showed that TRIM33 and P53 expressions are negatively correlated (Fig. S4A, B). Next, co-IP analysis was performed to further confirm the results of the IP/MS analysis, which showed that there was an interaction between TRIM33 and P53 (Fig. 4D, E). Therefore, we suspect that P53 may be the hub connecting TRIM33 and glycolysis. Consistent with our hypothesis, the expression of P53 increased and the expression of glycolysis-related genes decreased after TRIM33 knockdown, while silencing of P53 attenuated the downregulation of the glycolysis-related genes GLUT1, HK2, PKM2, and LDHA mediated by TRIM33 knockdown (Fig. 4F). Similarly, silencing P53 attenuated the downregulation of glucose uptake and lactate production mediated by TRIM33 knockdown (Fig. 4G, H), as well as the downregulation of ECAR (Fig. 4I). In order to prove that these processes are regulated by TRIM33 through the mediation of P53. TRIM33 was overexpressed in P53-silenced cell lines to study the changes in glycolysis products. The results showed that overexpression of TRIM33 in P53-silenced cell lines did not change glucose uptake, lactate production, and ECAR levels (Fig. S4C–F). Cell functional results showed that overexpression of TRIM33 in P53-silenced cell lines could not affect the proliferation and death of ESCC cells (Fig. S4G–L). In summary, the oncogenic activity of TRIM33 is P53-dependent.

**Fig. 5:**
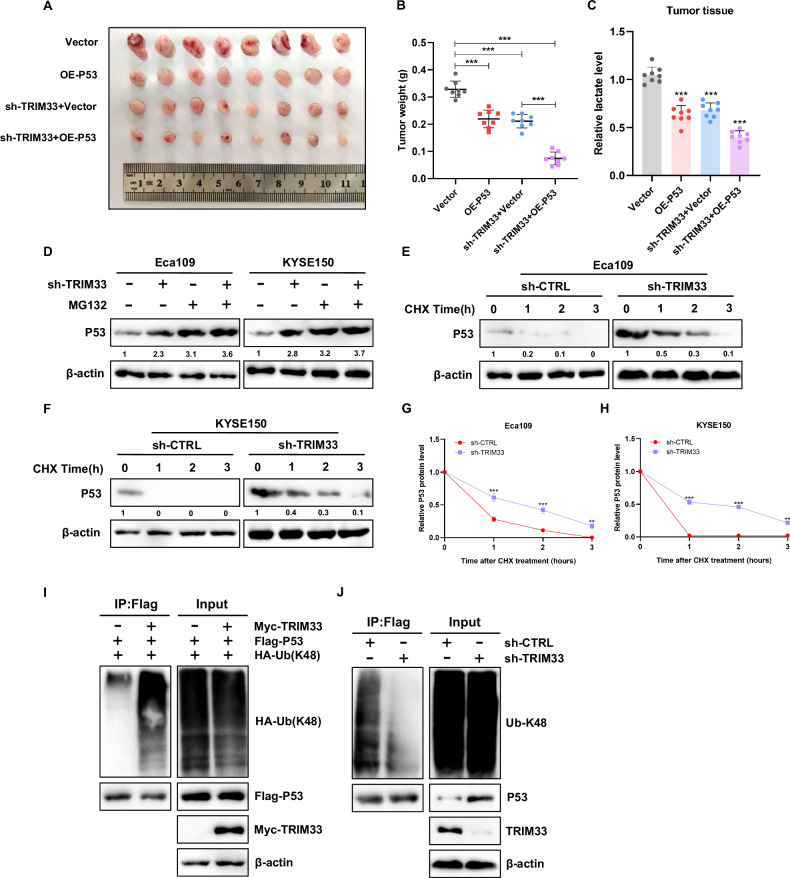
TRIM33 triggers P53 K48-linked ubiquitination and proteasome degradation. **A**, **B** The size and weight of subcutaneous xenografts of ESCC cells with different treatments. **C** Detection of lactic acid content in tumor tissues of nude mice. **D** In the presence of proteasome inhibitor MG132, Western blot analysis of the effect of P53 expression in ESCC cells affected by TRIM33. **E**–**H** Eca109 and KYSE150 cells stably interfering with TRIM33 and the control group were treated with cyclohexylamine (CHX) for 0, 1, 2, and 3 h respectively. The expression of P53 (**E**, **F**) was detected by Western blot, and the line graph of P53 expression (**G**, **H**) was further quantitatively analyzed. ImageJ was used to normalize actin for protein quantification. **I** HEK293 cells were transfected with FLAG-P53, Myc-TRIM33 and HA- ubiquitin K48-only (a ubiquitin construct in which all lysine residues except K48 were mutated into arginine; Therefore, only K48-linked Ub chain can be formed), and then immunoprecipitated with anti-labeled antibody, followed by immunoblotting with anti-K48-linked ubiquitin antibody. **J** HEK293 cells were lysed 48 h after transfection with sh-CTRL or shTRIM33, and then immunoprecipitated with P53 antibody, followed by immunoblotting with anti-K48-linked ubiquitin antibody.

To further verify the regulatory function of the TRIM33-P53 signal axis in vivo, we subcutaneously inoculated stable Eca109 cells transfected with Vector, OE-P53, shTRIM33+ vector, shTRIM33 + OE-P53 into nude mice to establish a mouse model of xenotransplantation. Analysis based on the size and weight of xenografts showed that the combination of the two groups significantly inhibited tumor growth compared to the silenced TRIM33 and overexpressed P53 groups (Fig. 5A, B). We also found that the expression level of lactic acid, the glycolysis product, was the lowest in shTRIM33 + OE-P53 nude mice tumors (Fig. 5C). From another point of view, this confirms that the TRIM33-P53 signal axis promotes tumor growth by enhancing the aerobic glycolysis of ESCC. In general, these results suggest that TRIM33 regulates P53 expression to regulate glycolytic activity.

### TRIM33 promotes P53 K48-linked polyubiquitin and proteasome pathway degradation

Because TRIM33 is an E3 ubiquitin ligase, it is suggested that TRIM33 may regulate the expression of P53 protein at the post-translational level. We further investigated whether TRIM33 affects the stability of P53. TRIM33 deletion increases P53 protein levels, which was attenuated by treatment with the proteasome inhibitor MG132 (Fig. 5D). These results indicate that TRIM33 regulates the level of P53 protein through its stability and promotes proteasome-dependent degradation of P53. Next, cycloheximide (CHX), an inhibitor of protein synthesis, was used to evaluate protein stability. The data showed that TRIM33 deletion significantly increased the half-life of endogenous P53 and inhibited its degradation of P53 protein in ESCC cells (Fig. 5E–H). K48-linked polyubiquitination is a classic protein degradation pathway. We further studied the effect of TRIM33 on the P53 K48-linked polyubiquitination. We transfected P53, K48, and TRIM33 plasmids into HEK293 cells. We immunoprecipitated P53 and detected its polyubiquitination of P53. Ubiquitin-based immunoprecipitation analysis showed that TRIM33 promoted P53 K48-linked polyubiquitination, whereas endogenous ubiquitin experiments showed that silencing of TRIM33 inhibited P53 K48-linked polyubiquitination (Fig. 5I, J). Therefore, TRIM33 promotes P53 K48-linked polyubiquitination and proteasomal degradation of P53.

### TRIM33 promotes aerobic glycolysis of ESCC through P53 K48-linked polyubiquitination mediated by the K351 site, thus promoting the growth of tumor cells

**Fig. 6:**
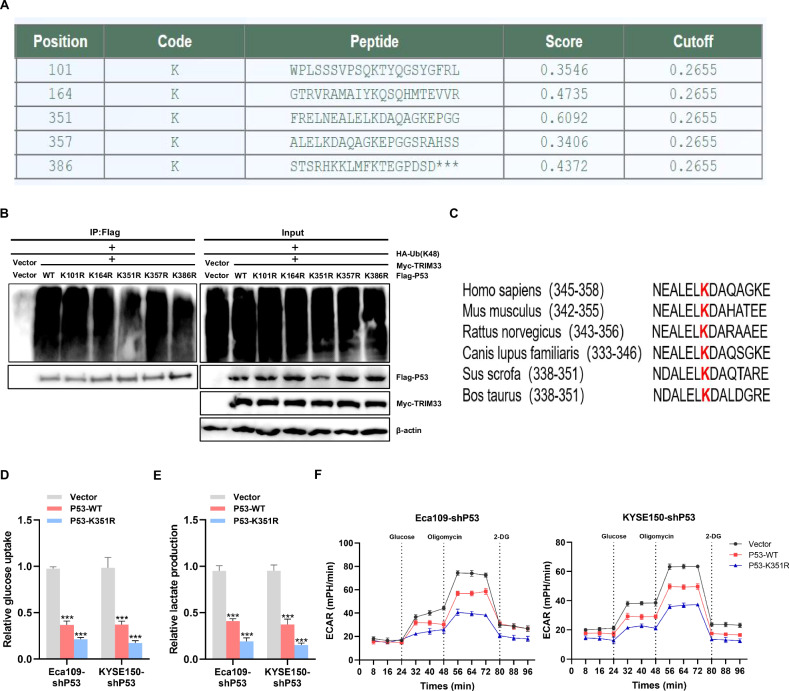
K351 site of P53 plays an important role in K48-linked ubiquitination and aerobic glycolysis of ESCC cells. **A** Prediction of ubiquitination site of P53 protein. **B** HEK293 cells were lysed 48 h after transfection with HA- ubiquitin K48-only and P53 mutant labeled with the FLAG shown, and then immunoprecipitated with anti-FLAG antibody, followed by immunoblotting with anti-HA antibody. **C** Sequence alignment around K351 residue of P53 homolog in different species. The red letter “K” indicates the lysine residue at the 351st position of P53. **D**–**F** The changes in glucose uptake (**D**), lactic acid production (**E**), and ECAR (**F**) in ESCC cells after the K351 site of P53 was mutated into arginine R were detected.

We predicted each potential lysine using GPS-Uber (http://gpsuber.biocuckoo.cn/) to identify the ubiquitination site of a specific protein [16], which was then used to identify the lysine residues related to P53 ubiquitination. The results showed that K101, K164, K351, K357, and K386 were all potential ubiquitination sites on the P53 protein (Fig. 6A). Ubiquitin-based immunoprecipitation analysis showed that, compared to K101, K164, K357, or K386, when lysine (K) 351 was replaced with arginine (R), ubiquitination was significantly reduced (Fig. 6B), indicating that K351 is a specific ubiquitination site of the P53 protein. Notably, the K351 site of P53 is highly conserved among different species (Fig. 6C). This strongly indicates that K351 is a key site for mediating P53 ubiquitination. In addition, when K351 was replaced by arginine (R), compared to wild-type P53, glucose uptake and lactic acid production decreased (Fig. 6D, E), and ECAR was downregulated (Fig. 6F). In summary, K351 plays an important role in P53 K48-linked polyubiquitination and aerobic glycolysis in ESCC cells.

**Fig. 7:**
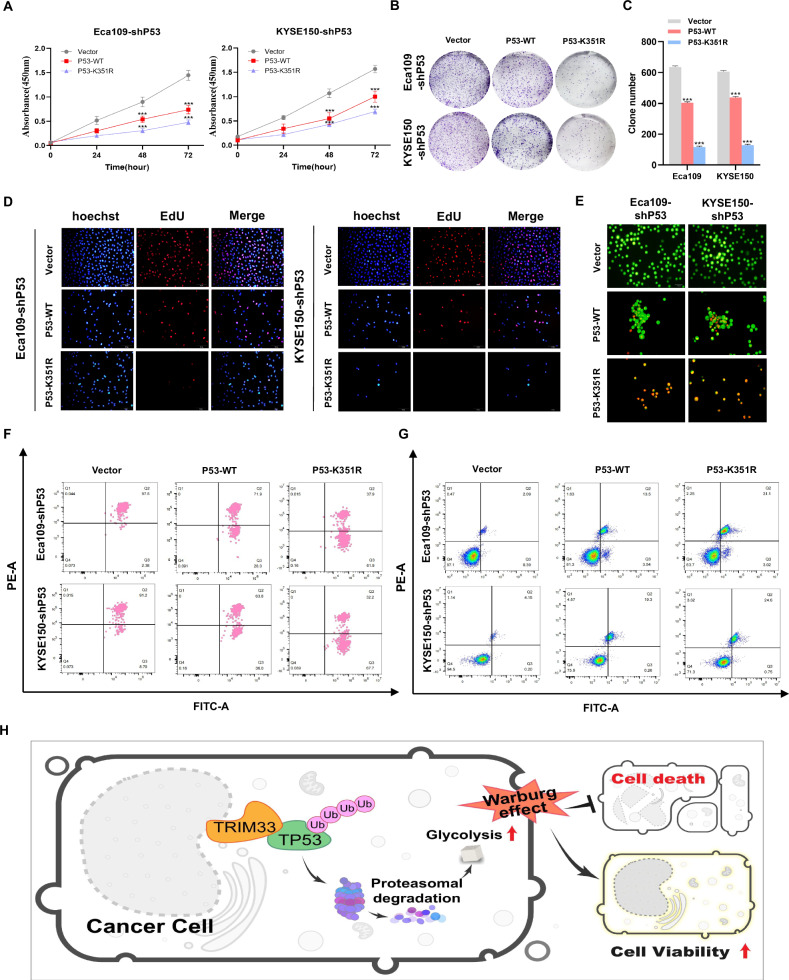
K351 site of P53 is the key site affecting the proliferation and death of ESCC cells. **A**–**D** Wild-type P53 and P53-K351R mutant plasmids were transfected into ESCC cell lines with stable P53 knockdown, and the effects of K351 mutation on the proliferation of ESCC cells were detected, as shown in CCK-8 assay (**A**), colony formation test (**B**, **C**), and EdU assay. Scale, 50 μm (**D**). (**E**–**G**) Analyze the influence of the K351 mutation of P53 protein on the membrane potential of Eca109 and KYSE150 cells by JC-1 assay (**F**). AO staining and flow cytometry were used to evaluate the effect of K351 mutation on cell death (**E**, **G**). Scale, 50 μm. **H** The mechanism map shows that TRIM33 promotes ESCC tumor proliferation and glycolysis by ubiquitin-induced degradation of P53.

To explore the influence of the K351 site of P53 on the biological function of ESCC cells, we used lentiviral shRNAs to silence endogenous P53 expression in Eca109 and KYSE150 cells. On this basis, we divided them into three groups and transfected the Vector, P53-WT and P53 mutant K351R plasmids. The results of the CCK-8 cell viability assay, colony formation assay, and EdU proliferation assay showed that overexpression of P53-WT inhibited ESCC cell growth and colony formation, while the P53 mutant K351R significantly inhibited cell proliferation (Fig. 7A–D). We evaluated the effect of K351 on cell death. The results of membrane permeability JC-1 staining showed that, compared to the control group, P53-WT reduced the mitochondrial membrane potential, while the K351R mutant had the lowest mitochondrial membrane potential (Fig. 7F). The results of AO staining and flow cytometry showed that the number and proportion of P53-K351R apoptotic cells were significantly higher than those in the Vector and P53-WT groups (Fig. 7E, G). This suggests that K351 of P53 is the key site affecting the aerobic glycolysis, proliferation, and death of ESCC cells. In summary, these results reveal the molecular mechanism by which TRIM33 controls ESCC tumor proliferation and glycolysis through the ubiquitination of P53 (Fig. 7H).

## Discussion

E3 ubiquitin ligase TRIM33 is a member of the TRIM family, also known as TIF1 γ, RFG7, PTC7, or Ectodermin. Proteins of this family have been reported to play important roles in cancer progression [17]. However, the role of TRIM33 in tumors remains controversial. It may be both carcinogenic and tumor-suppressive. For example, TRIM33 is highly expressed in colorectal cancer, and its expression level is related to tumor stage [18, 19]. Increased expression of TRIM33 in colorectal cancer is associated with the loss of Smad4 and indicates a poor prognosis in patients with colorectal cancer [19]. In non-small cell lung cancer (NSCLC), circPTK2 inhibits epithelial-mesenchymal transformation and metastasis induced by TGF β by controlling TRIM33 [20]. TRIM33 also drives prostate tumor growth by stabilizing Skp2-mediated androgen receptor degradation [11]. However, it also plays a tumor-suppressive role in glioblastoma. TRIM33 inhibits tumor cell proliferation and tumorigenesis by degrading nuclear β-catenin [13]. In hepatocellular carcinoma, the CpG island of the TRIM33 promoter is hypermethylated, and its expression is decreased. At the same time, the decreased expression of TRIM33 is an independent and significant risk factor for recurrence and survival after radical resection of liver cancer [21]. Interestingly, even in the same type of cancer, TRIM33 may be carcinogenic or tumor-suppressive. As reported in the literature, the overexpression of FOXM1 in breast cancer can interact with Smad3/Smad4 to inhibit the binding of TRIM33 and Smad4, thus preventing its ubiquitination, thus weakening the inhibition of TRIM33 on transforming growth factor-β signal, thus promoting breast cancer metastasis [15]. However, another study reported that the expression of TRIM33 was increased in 35.9% of patients with breast cancer, and its expression was associated with younger age, estrogen receptor (ER) negativity, and tumor diameter >2 cm. In addition, the overexpression of TRIM33 is related to poor prognosis in patients with breast cancer [22]. This further indicates that TRIM33 has a unique cell-type, background, and functional specificity, even in the same cancer type. Because the role of TRIM33 in ESCC remains unclear, we attempted to describe the characteristics of TRIM33 in ESCC.

In the current study, we report for the first time the role and mechanism of action of TRIM33 in ESCC progression. TRIM33 is highly expressed in ESCC tissues and cell lines and is associated with adverse clinical outcomes. In vivo and in vitro experiments have revealed that TRIM33 promotes the proliferation of ESCC cells, inhibits cell death, promotes tumor growth in nude mice, and plays a carcinogenic role in the occurrence and development of ESCC. Considering the carcinogenic effects of TRIM33, we studied the molecular mechanisms underlying the effects of TRIM33 on ESCC. We combined IP with LC-MS to determine the potential target proteins of TRIM33 and their functions in ESCC. Bioinformatics analysis, loss of function, and acquisition of function methods have proven the role of TRIM33 in regulating glycolysis in ESCC cells, which has not been previously reported.

Increased glucose metabolism and reprogramming to aerobic glycolysis are signs that cancer cells meet the needs of cell proliferation and metabolism. Many metabolic pathway disorders have been described in the pathogenesis and progression of ESCC; however, their specific molecular mechanisms remain unclear. Our study confirmed that P53 is the target protein of TRIM33 and that the two proteins interact with each other. It has been reported that P53 can inhibit glycolysis at many steps [23], but the exact target genes responsible for glucose metabolism must be enriched [24, 25]. In this study, TRIM33 binds to P53 to inhibit its expression of P53 and promote the expression of the downstream glycolysis target genes GLUT1, HK2, PKM2, and LDHA. Loss-of-function and salvage experiments showed that TRIM33 promotes aerobic glycolysis in ESCC by inhibiting P53. Therefore, the TRIM33-P53 axis plays a key role in cancer cells by linking glycolysis to cell proliferation.

The expression and function of P53 are regulated by post-translational modifications [26, 27]. Among these modifications, ubiquitination, which is closely related to protein stability and degradation, has attracted considerable attention in cancer research. Ubiquitination, mediated by members of the E3 ligase TRIM family, is a key step in the degradation of P53 protein. TRIM31 deletion promotes breast cancer progression by regulating K48- and K63-linked ubiquitination of P53 [28]. TRIM32 ubiquitin P53 decreases the activity of P53 and promotes tumorigenesis [29]. TRIM45 stabilizes P53 via K63-linked ubiquitination, thereby inhibiting glioma progression [30]. TRIM67 inhibits the degradation of P53 through its ubiquitin ligase MDM2, thus inhibiting the occurrence of colorectal cancer [31]. In this study, we found that TRIM33 promotes K48-linked polyubiquitin and proteasomal degradation of P53. Further studies have shown that TRIM33 promotes the growth of tumor cells by promoting aerobic glycolysis in ESCC through K48-linked polyubiquitination of P53 mediated by the K351 site of P53. This is the first study to show that TRIM33-mediated post-translational modifications play an important role in aerobic glycolysis and tumorigenesis. In summary, these findings further describe the detailed mechanisms of TRIM33-mediated P53 ubiquitination and cancer metabolic remodeling.

In summary, our findings emphasize the importance of the TRIM33-mediated post-translational regulation of P53 in aerobic glycolysis and ESCC tumor progression. The expression of TRIM33 can be used as an early biomarker to evaluate the risk of malignant transformation and as an intervention target for ESCC, providing an option for cancer treatment targeting glucose metabolism.

## Materials and methods

### Cell lines and human tissue specimens

Human esophageal epithelial cells (HEEC) is purchased from the American Type Culture Collection (ATCC). ESCC cell lines (Eca109, KYSE150, and TE-1) were purchased from the Institute of Biochemistry and Cell Biology of the Chinese Academy of Sciences, Eca109 is P53 wild-type cell. KYSE150 and TE-1 are P53 mutant cells. All cells were cultured in a constant temperature incubator containing 10%FBS (Gibco) and 1% penicillin-streptomycin (Solarbio) in a constant temperature incubator containing 5%CO_2_ at 37 °C. Specimens were collected from the First Affiliated Hospital of the Medical College of Shihezi University, China. Eighty paraffin-embedded ESCC tissue specimens and 79 adjacent noncancerous tissue specimens were confirmed pathologically. The study was conducted in accordance with the ethical guidelines of the Declaration of Helsinki and approved by the Hospital Ethics Committee, and all recruited subjects were enrolled with written informed consent.

### Reagents and antibodies

Cycloheximide (CHX) was purchased from Sigma Aldrich. MG132 was purchased from Selleck Chemicals. Lipofectamine 2000 reagent (Invitrogen, 11668-027). The main antibodies were as follows: anti-Flag-tag (66008-4-Ig, Proteintech), anti-HA-tag (81290-1-RR, Proteintech), anti-Myc-tag (16286-1-AP, Proteintech), anti-TRIM33 (16286-1-AP, Sigma Aldrich), anti-TRIM33 (Ab47062, Abcam), anti-P53 (Ab26, Abcam), anti-Ubiquitin-K48 (Ab140601, Abcam), anti-GLUT1 (21829-1-AP, Proteintech), anti-HK2 (22029-1-AP, Proteintech), anti-PKM2 (15822-1-AP, Proteintech), anti-LDHA (19987-1-AP, Proteintech), anti-β-actin (66009-1-Ig, Proteintech), anti- Lamin B1 (12987-1-AP, Proteintech), goat anti-mouse/rabbit IgG (ZB-2305/2301, ZSGB), TRITC goat anti-mouse IgG (ZF-0313, ZSGB), FITC goat anti-rabbit IgG (ZF-0311, ZSGB).

### Plasmids and shRNAs

The pEnCMV-TRIM33-Myc, pCMV-P53-FLAG, and P53 point mutation plasmids (K101R, K164R, K351R, K357R, and K386R) were constructed using molecular cloning technology. HA-UB-K48 was purchased from MiaoLing Biology, and lentivirus packaging plasmids (pMD2.G and psPAX2) were purchased from Addgene. The shRNA target sequences and PCR primer sequences are listed in Table S1.

### Quantitative real-time PCR (qRT-PCR)

Total RNA was extracted using TRIzol reagent (Thermo Fisher Scientific) and reverse-transcribed into cDNA. Using cDNA as a template, SYBR Premix Ex Taq and primers were added, and an ABI 7500 detection system (Applied Biosystems) was used to analyze mRNA expression.

### Immunohistochemistry (IHC) and immunofluorescence (IF)

Paraffin-embedded human or mouse tumor sections were stained with IHC. The slices were dewaxed, rehydrated with xylene, washed with gradient alcohol and PBS, repaired with antigen, blocked by endogenous peroxidase with TBS/H_2_O_2_, and then incubated overnight in primary antibodies at 4 °C. The next day, the slices were rewarmed at room temperature for 30 min, incubated with secondary antibodies at room temperature for 30 min, stained with DAB, re-stained with hematoxylin, and further analyzed using ImageJ.

The slides were fixed with 4% paraformaldehyde for 15 min, washed with PBS, and soaked in 0.5%TritonX-100 for 10 min. The serum was sealed for 30 min at room temperature, and the first antibody was incubated overnight at 4 °C. The next day, the cells were incubated with a fluorescent secondary antibody for 1 h, and the nuclei were stained with DAPI, and photographed using a fluorescence microscope (OlympusBX51, Japan).

### Nuclear and cytoplasmic extraction

Cytoplasm and nucleus proteins were separately extracted using Minute Cytoplasmic and Nuclear Extraction Kits for Cells (SC-003, Invent Biotechnologies). The sample is processed according to the instructions of the reagent manufacturer.

### Western blot and Co-IP assay

The extracted protein samples were electrophoretic, the protein molecules on the gel were electrically transferred to the PVDF membrane (Millipore), soaked in the blocking solution (5% skim milk) for 2 h, then the first antibody was incubated overnight at 4 °C, and the membrane was placed in the second antibody the next day and incubated at room temperature for 2 h. A chemiluminescent HRP substrate (Thermo Fisher Scientific) was used to develop the film. For the Co-IP experiment, the necessary cell lysates were prepared and incubated with corresponding antibodies and Protein A/G beads (Sigma Aldrich) at 4 °C for the night. The following day, the beads were washed with PBST and boiled at 100 °C for 10 min. Immunoprecipitates were collected and analyzed by western blot.

### Molecular docking (protein–protein docking)

The target protein TRIM33 and P53 protein were searched in Uniprot database (https://www.uniprot.org/), and the corresponding Uniprot ID were Q9UPN9 and P04637, respectively. The 3D structures of the two proteins were docked with the Protein-Protein docking (Piper) module in Schrödinger.

### CCK-8 assay

Cytotoxicity and proliferation were analyzed using CCK-8 and Dojindo. Tumor cells were cultured in 96-well plates. About 0, 24, 48, and 72 h, the CCK-8 reagent was added, and the absorbance of the solution was measured at 450 nm.

### Colony formation assay

The cells were inoculated into a six-well plate according to the experimental group. After 2 weeks of culture, the cells were fixed with 4% paraformaldehyde solution for 15 min, stained with 0.1% crystal violet solution, washed with PBS, and the number of colonies was counted using ImageJ.

### 5-Ethynyl-2′-deoxyuridine (EdU) staining

The cells (1 × 10^5^ cells/well) were seeded in 12-well plates. According to the manufacturer’s plan, the cells were labeled with an EdU kit (KGA337, KeyGen Biotechnology, China) and photographed under a fluorescence microscope.

### TUNEL staining

The pretreated cells were fixed in 4% paraformaldehyde for ~30 min and washed three times with PBS. TUNEL detection solution (Beyotime Biotechnology, China) was added to each well and incubated at 37 °C for 1 h. Images were collected.

### JC-1 assay

The cells were suspended in 1 mL medium with a concentration of about 1 × 10^6^ cells/mL, and 10 μL JC-1 (Thermo Fisher Scientific) was added and cultured at 37 °C and 5% CO_2_ for 30 min. The cells were precipitated by centrifugation and re-suspended by adding 500 μL PBS to each test tube and analyzed by flow cytometry (Beckman CytoFLEX).

### Acridine orange (AO) staining

Add 4 μL AO/EB dye to 100 μL cell suspension and mix it evenly. A drop of the above mixture was placed on a glass sheet, covered, and the staining results were observed with a fluorescence microscope.

### Flow cytometry of apoptosis

Annexin V-FITC (5 μL) and PI staining solution (10 μL) were added to each sample, and the cells were analyzed using flow cytometry (Beckman CytoFLEX).

### Liquid chromatography–mass spectrometry (LC/MS)

Firstly, dithiothreitol (Sigma Aldrich) solution was added to the sample to make the final concentration of 10 mmol/L, which was reduced in a 56 °C water bath for 1 h. Add iodine acetamide solution (Sigma Aldrich) to make the final concentration of 55 mmol/L, avoiding light reaction 40 min. The peptides were dissolved in a sample solution (0.1% formic acid) and subjected to mass spectrometry. The mass spectrometry conditions were as follows: mobile phase A was 0.1% formic acid aqueous solution, mobile phase B was 80% ACN/0.1% formic acid aqueous solution, the flow rate was 600 nL/min, and the detection time for each group of samples by liquid chromatography (Easy-nLC1200, Thermo Fisher Scientific) was 66 min.

### Glucose uptake assay and lactate assay

The levels of glucose and lactic acid in the culture medium were measured by colorimetric glucose uptake assay kit (AAT Bioquest) and l-lactic acid assay kit (AAT Bioquest), respectively. The sample is processed according to the instructions of the reagent manufacturer, and then the sample is detected under Ex/Em = 570/610 nm using the TECAN SPARK enzyme labeling instrument.

### Glycolysis stress test

The extracellular acidification rate (ECAR) and oxygen consumption rate (OCR) of the ESCC cells was measured using a hippocampal XF24 extracellular flux analyzer (Agilent SeaHorse Bioscience) according to the manufacturer’s protocol. Briefly, the culture medium was inoculated at a density of 5 × 10^4^ cells/well, and cell viability was determined using the XF cell glycolysis test kit (Agilent).

### Polyubiquitination assay

To directly detect ubiquitinated P53 of K48 in the cell extract, HEK293 cells were transfected with K48-Ub or experimental protein plasmids. After 36 h, the total protein was extracted, and the cell lysate was incubated with 40 μL Protein A/G beads (Sigma Aldrich) and antibody at 4 °C overnight. The immunoprecipitate was collected and analyzed by western blot.

### Xenograft tumor model

Animal studies were approved by the Ethics Committee of The First Affiliated Hospital, Shihezi University School of Medicine. All animal experiments complied with the National Research Council Guide for the Care and Use of Laboratory Animals. Forty-eight 4-6-week-old female BALB/c nude mice were purchased from the Institute of Experimental Animals, Chinese Academy of Medical Sciences, Beijing. The weights of nude mice were measured before inoculation. According to the experimental group, eight nude mice in each group, the stably transfected cells were digested in 0.25% trypsin, centrifuged with 2000 rpm for 10 min, and re-suspended in 100 μL DMEM medium (containing about 3 × 10^6^ cells). Then, subcutaneously injected into the nude mice, tumor formation was monitored every day in nude mice, and tumor size and weight of nude mice were measured every 3 days. After 6 weeks, the nude mice were sacrificed and imaged, and the tumor tissues were preserved. The longest diameter (a) and the shortest diameter (b) were measured. Additionally, the volume of the tumor was measured using Vernier calipers to calculate the size of the tumor (V) according to the formula V (mm^3^) = 1/6 πab^2^. Tumor tissues were reserved for follow-up experiments.

### Statistical analysis

SPSS analyses were performed using IBM SPSS Statistics for Windows, version 27.0. An independent samples t-test was used to compare the differences between the two groups. A one-way analysis of variance (ANOVA) was used to compare indicators between different groups. Statistical significance was set at *p* < 0.05. significant. All graphs were generated using GraphPad Prism 9.0 (GraphPad Software, CA, USA). Significance is indicated by *P < 0.05, **P < 0.01, and ***P < 0.001.

## Supplementary information

Supplementary figures and table

Original Western Blot

## Data Availability

We are committed to sharing research data upon reasonable request. All data and materials generated in this study are available from the corresponding author.
