# Supplementary material for: TRIM33 promotes glycolysis through regulating P53 K48-linked ubiquitination to promote esophageal squamous cell carcinoma growth
Source: Cell Death Dis. 2024 Oct 10;15(10):740. doi: 10.1038/s41419-024-07137-z (PMC11467421; doi:10.1038/s41419-024-07137-z)
Supplement: Supplementary file 1 — Supplementary figures and table [file 41419_2024_7137_MOESM1_ESM.pdf]

Supplementary Figure 1

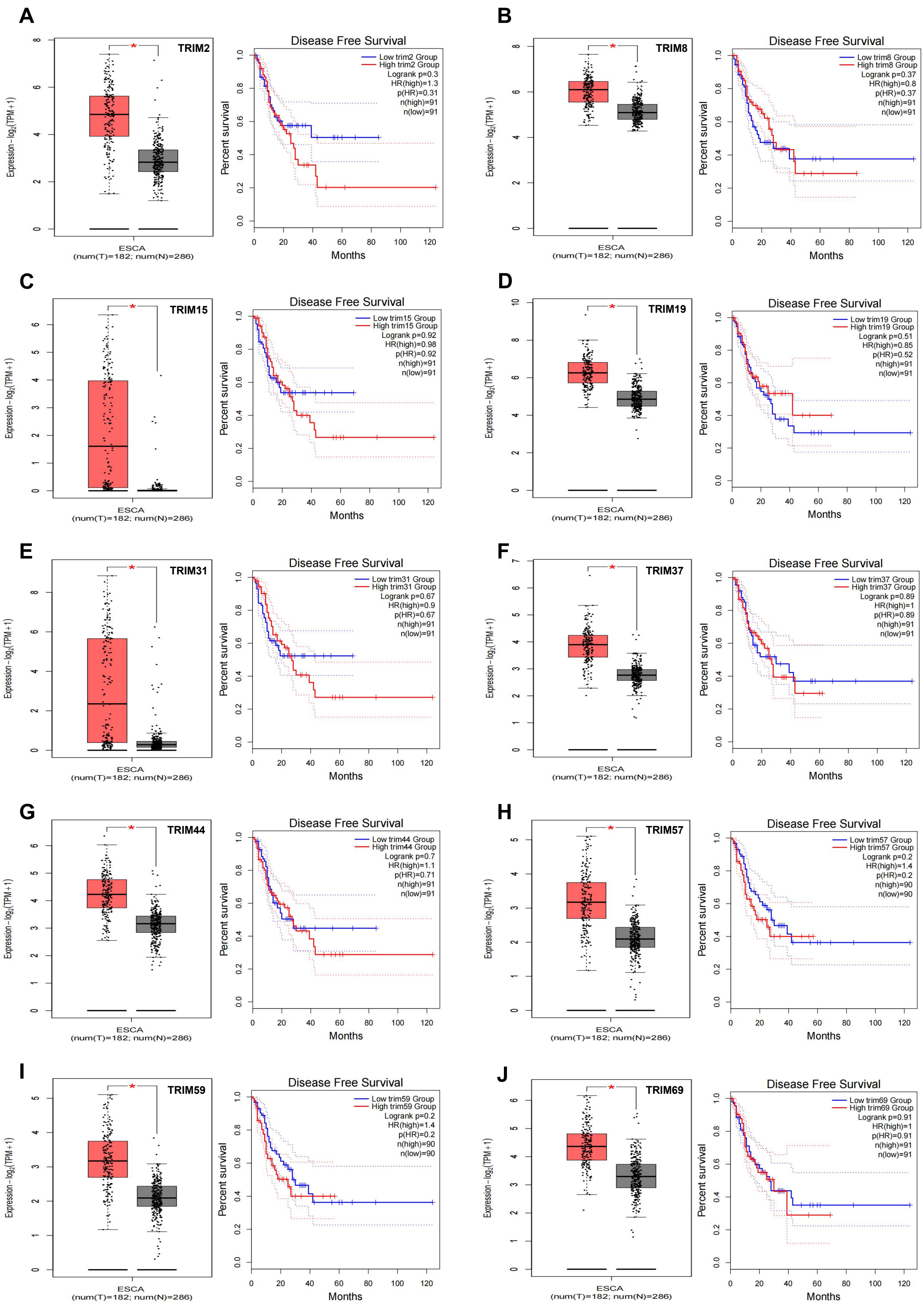

Supplementary Fig. 1. The expression difference of TRIM family genes in esophageal cancer and non-cancer tissues, and the comparison of DFS between patients with high and low expression of TRIM protein

(A) TRIM2. (B) TRIM8. (C) TRIM15. (D) TRIM19. (E) TRIM31. (F) TRIM37. (G) TRIM44. (H) TRIM57. (I) TRIM59. (J) TRIM69. HR hazard ratio, DFS Disease free survival.

Supplementary Figure 2

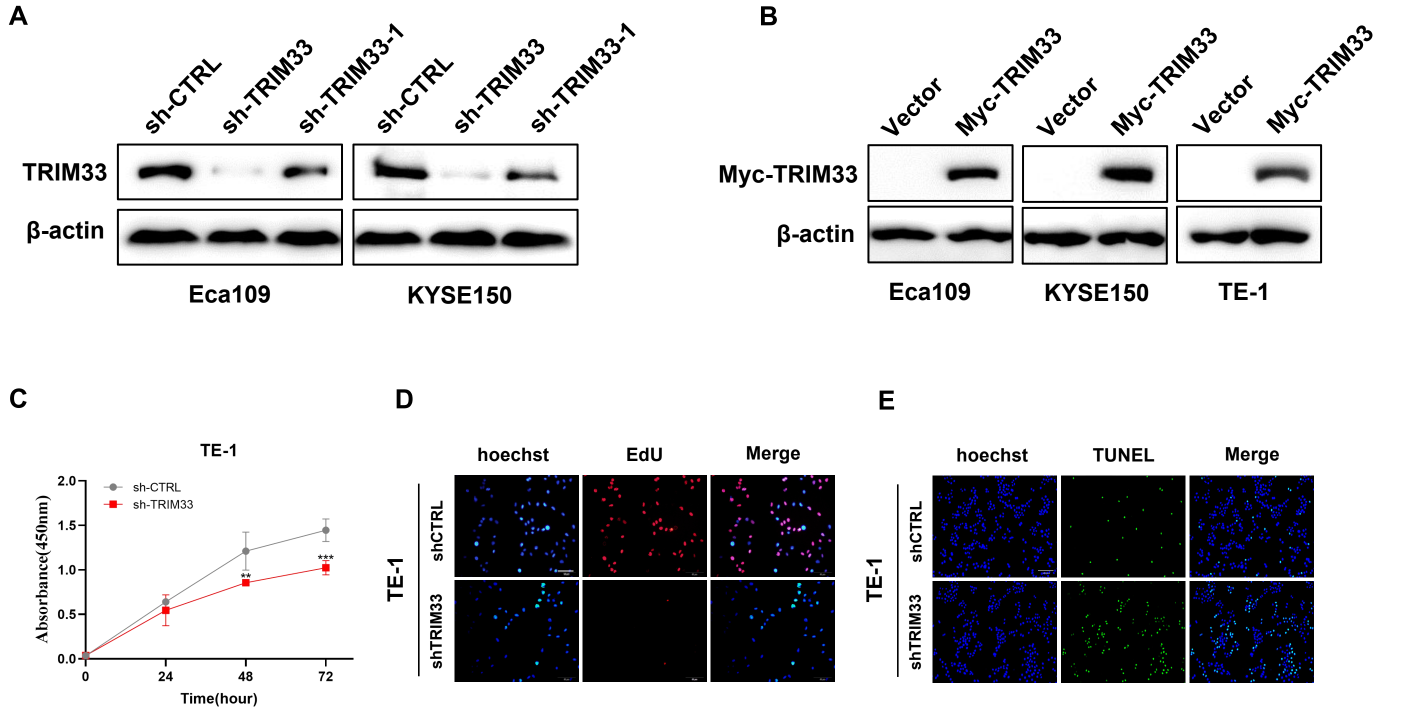

Supplementary Fig. 2. The expression efficiency of knock-down or over-expression of TRIM33 in ESCC cells was detected and function of TRIM33 in TE-1 cells.

(A) Western blot analysis of the expression level of TRIM33 in sh-TRIM33 and sh-CTRL cells. (B) Western blot analysis of the tag protein after transfection of Myc-TRIM33 plasmid. (C and D) Knockdown of TRIM33 by shRNA suppresses the proliferation of TE-1 cell as revealed by the CCK-8 assay (C) and EdU assay. Scale bars, 50 μm (D). (E) TRIM33 knockdown increased the number of TUNEL-positive death cells. Scale bars, 100 μm.

Supplementary Figure 3

A

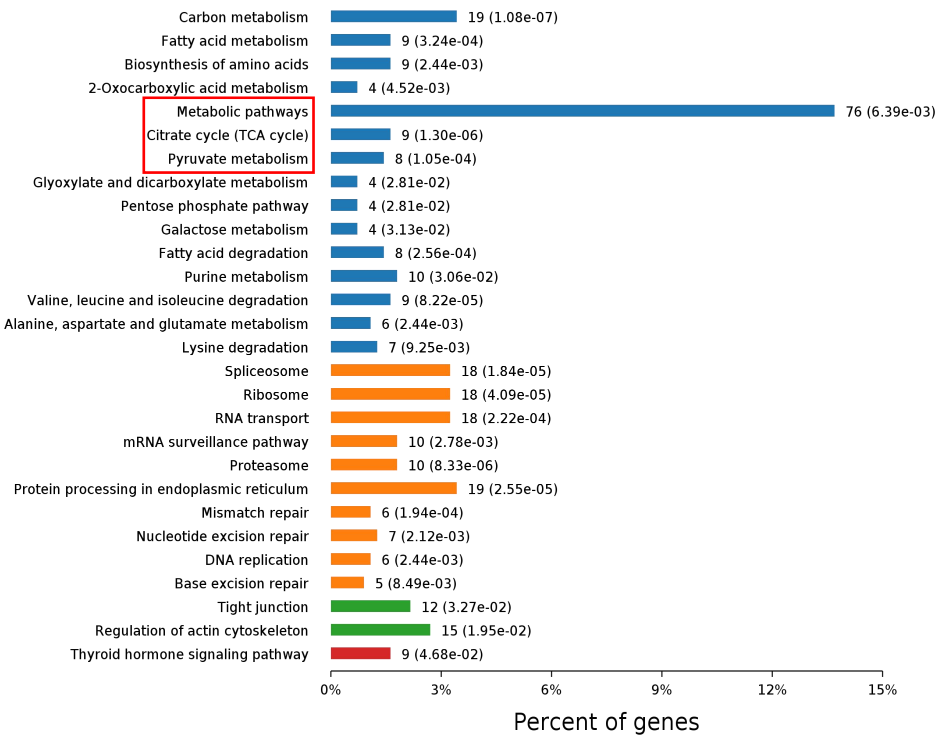

B

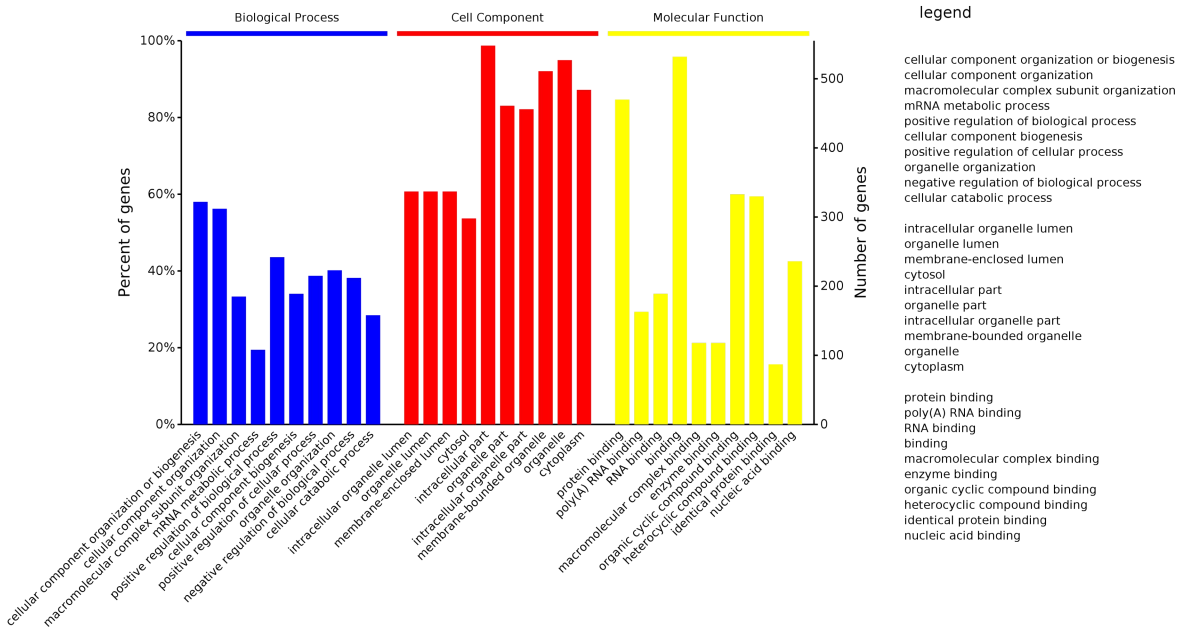

Supplementary Fig. 3. LC-MS/MS data analysis of potential target proteins in TRIM33

(A) the proportion of potential target protein of TRIM33 in KEGG pathway enrichment categories. (B) GO enrichment results statistics.

Supplementary Figure 4

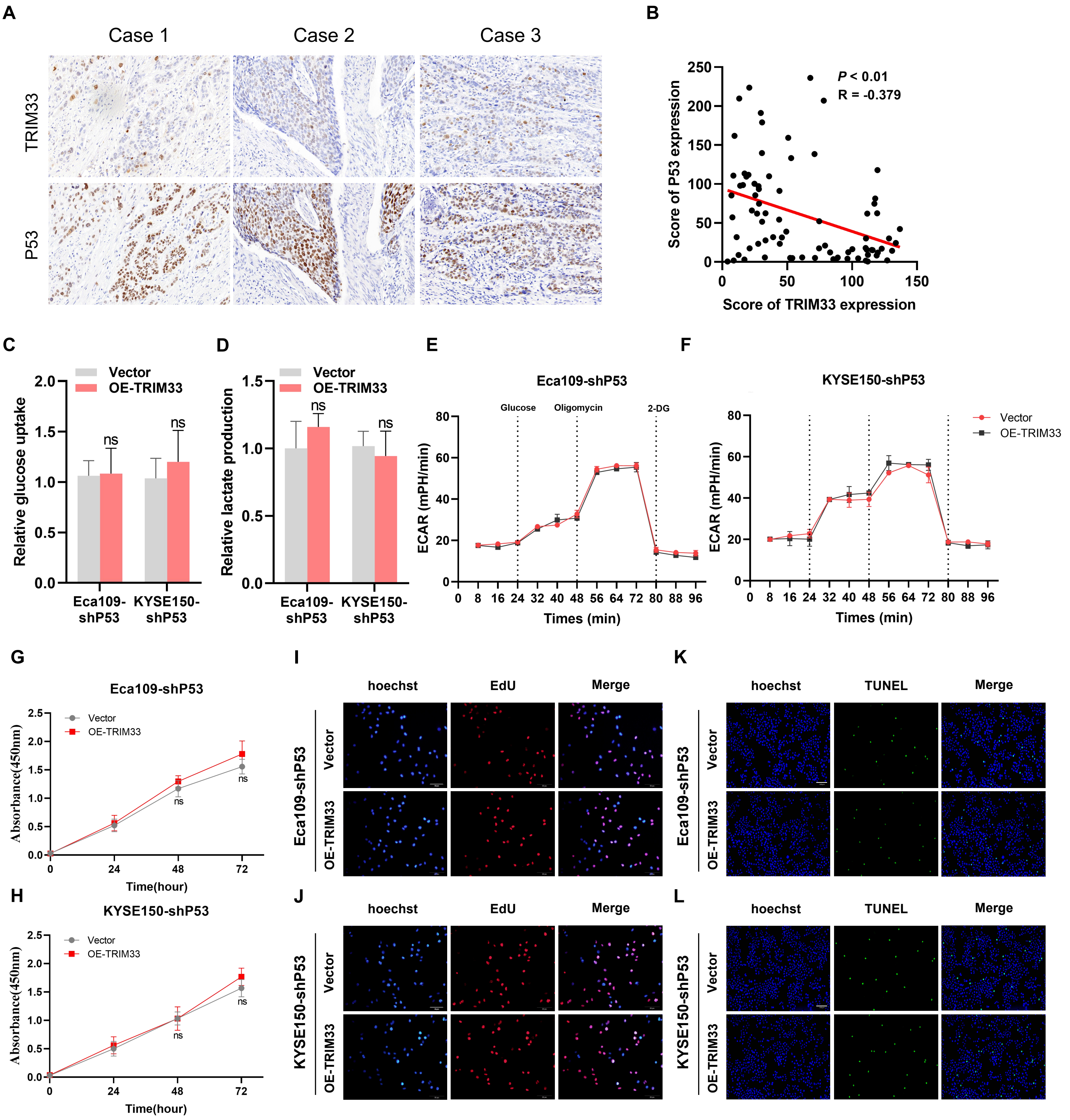

Supplementary Fig. 4. TRIM33 and P53 expressions are negatively correlated, and the oncogenic activity of TRIM33 is TP53 dependent

(A) Representative IHC staining comparison images (40×) of TRIM33 and P53 in the same tissue sample. (B) The correlation analysis between TRIM33 and P53 were carried out. (C-F) The glucose uptake rates (C), Lactic acid production (D), ECAR (E-F) were detected by overexpression of TRIM33 in cell lines silenced for TP53. (G-J) The influence of TRIM33 on the proliferation ability of P53-silenced ESCC cells were measured by the CCK-8 assay (G and H) and EdU assay. Scale bars, 50 μm (I and J). (K and L) Detection of the number of TUNEL-positive cells overexpressing TRIM33 in P53-silenced cell lines. Scale bars, 100 μm.

.

Supplementary Table 1 Primer sequence information

Primer name	Sequence
sh-CTRL	CAACAAGATGAAGAGCACCAA
sh-TRIM33	GTACTAGTTGTGAAGACAATG
sh-TRIM33-1	TACTTTCCAGTTGCGTCATAT
sh-P53	CGGCGCACAGAGGAAGAGAAT
qpcr-TRIM33-F	GGAGTGCTTGCATGTTGAG
qpcr-TRIM33-R	CCAATTCACTTTCTAGATGCAGG
qpcr-GAPDH-F	GAAGGTGAAGGTCGGAGTCA
qpcr-GAPDH-R	TTGAGGTCAATGAAGGGGTC
