# Supplementary figures and images for: TRIM33 promotes glycolysis through regulating P53 K48-linked ubiquitination to promote esophageal squamous cell carcinoma growth
Source: Cell Death Dis. 2024 Oct 10;15(10):740. doi: 10.1038/s41419-024-07137-z (PMC11467421; doi:10.1038/s41419-024-07137-z)

Figure 1

I

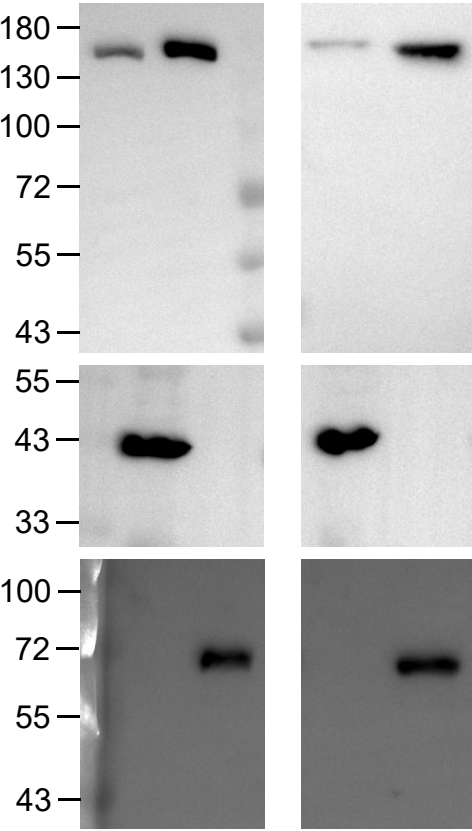

Figure 4

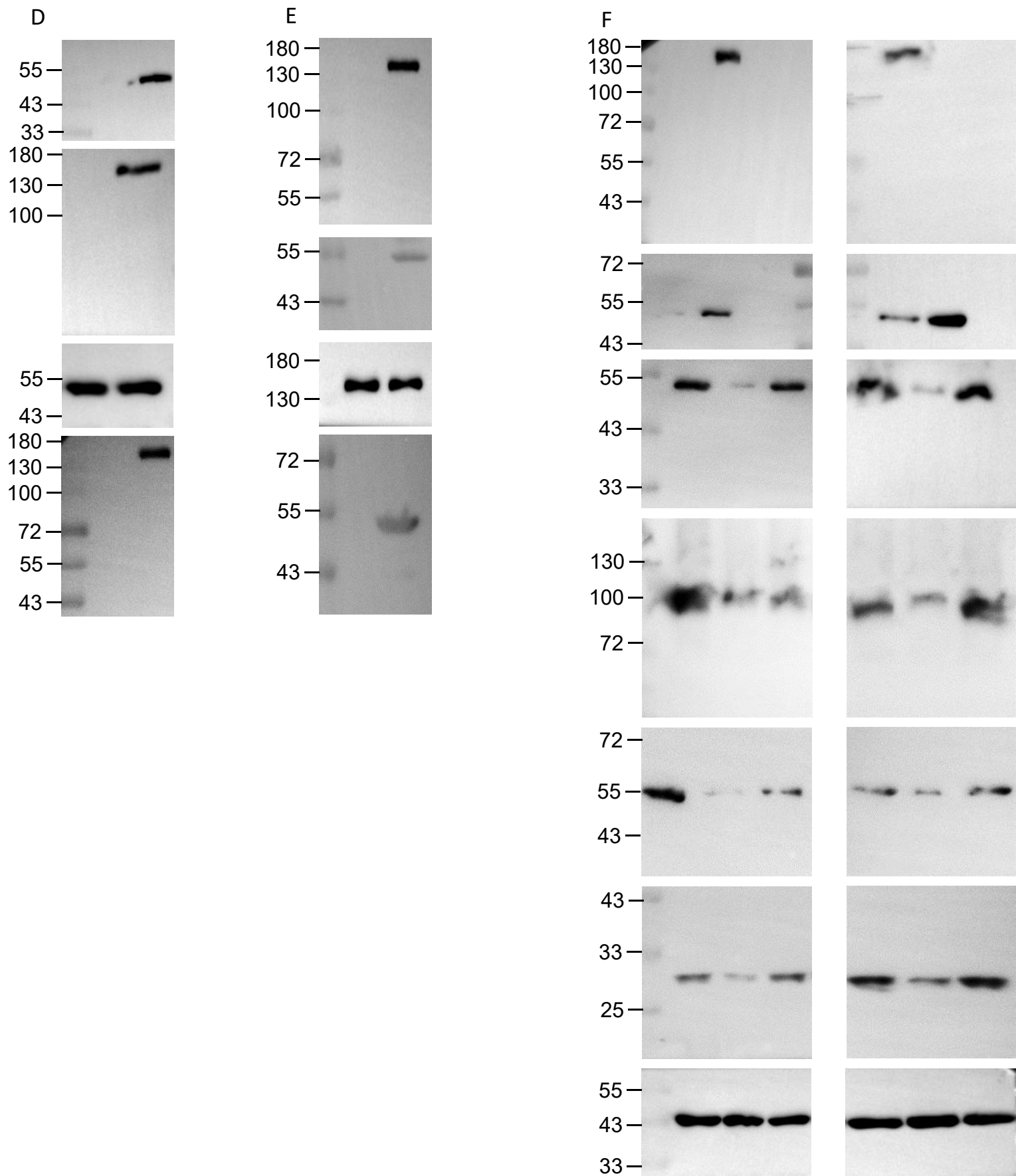

Figure 5

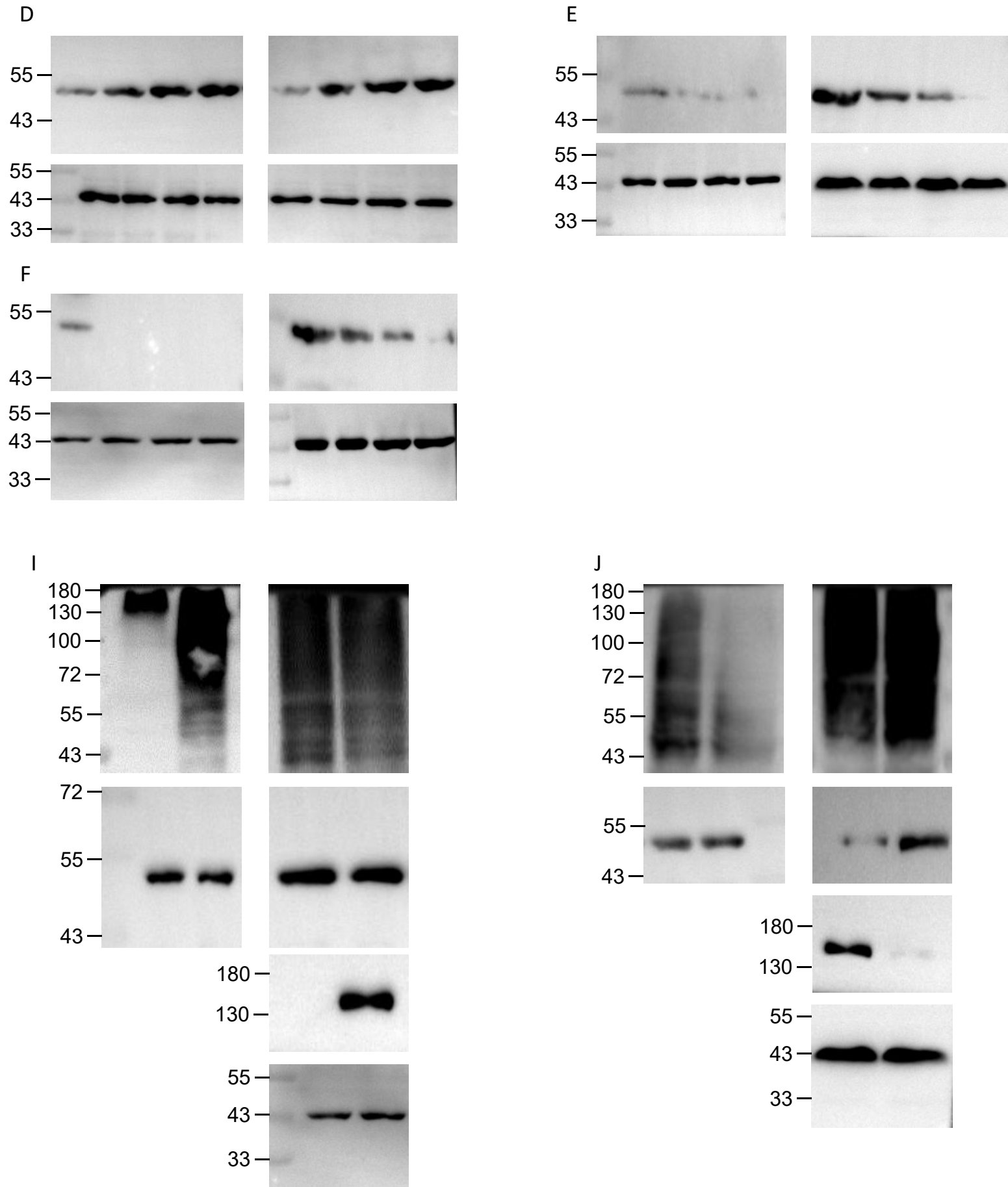

Figure 6

B

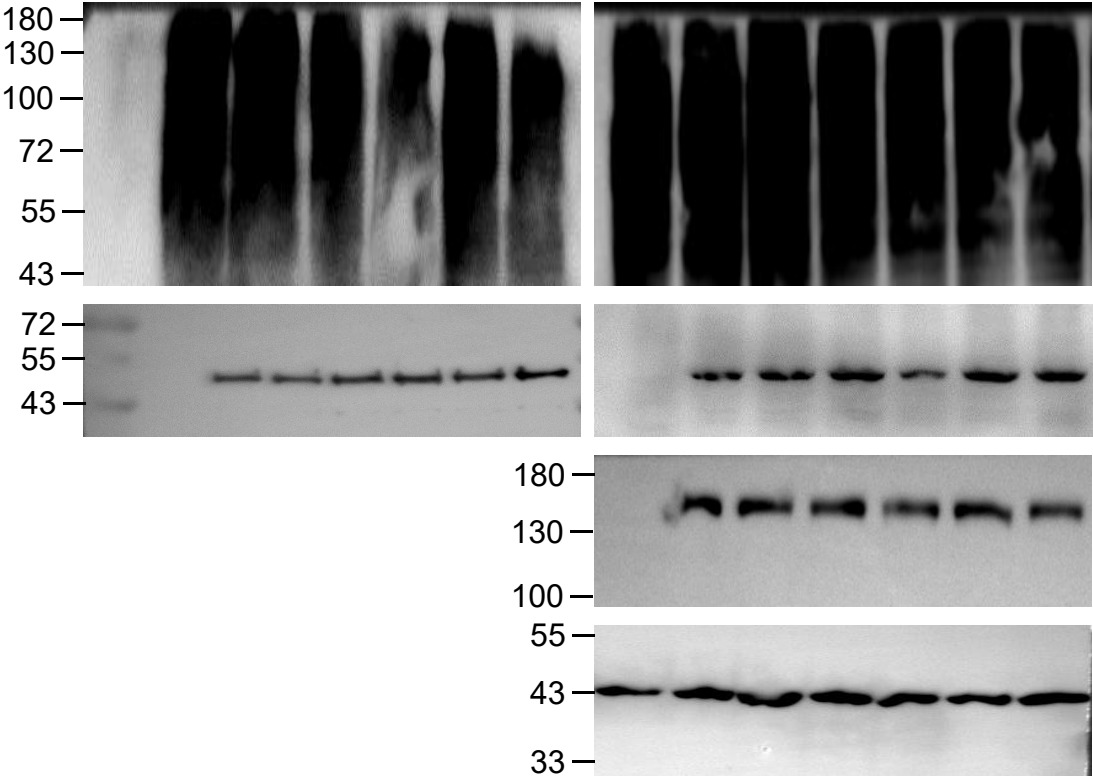

Supplementary Figure 2

A

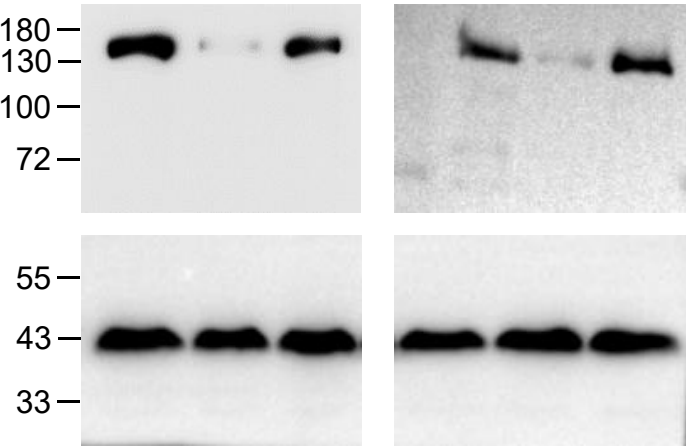

B

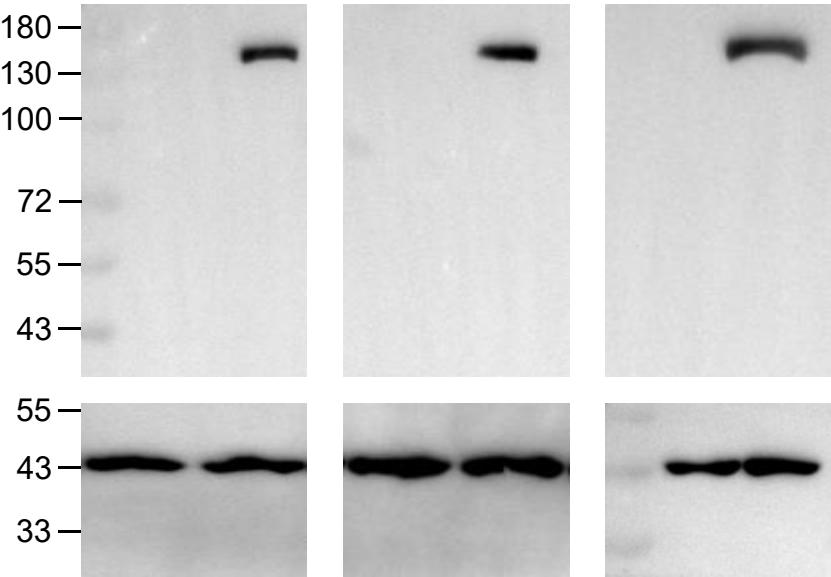

Supplement: Supplementary file 2 — Original Western Blot [file 41419_2024_7137_MOESM2_ESM.pdf]
